# Workplace Social Capital and Adherence to Antihypertensive Medication: A Cohort Study

**DOI:** 10.1371/journal.pone.0024732

**Published:** 2011-09-09

**Authors:** Tuula Oksanen, Ichiro Kawachi, Anne Kouvonen, Etsuji Suzuki, Soshi Takao, Noora Sjösten, Marianna Virtanen, Jaana Pentti, Jussi Vahtera, Mika Kivimäki

**Affiliations:** 1 Finnish Institute of Occupational Health, Helsinki, Finland; 2 Department of Society, Human Development and Health, Harvard School of Public Health, Boston, Massachusetts, United States of America; 3 Warsaw School of Social Sciences and Humanities, Wroclaw Faculty, Wroclaw, Poland; 4 Department of Epidemiology, Okayama University Graduate School of Medicine, Dentistry and Pharmaceutical Sciences, Okayama, Japan; 5 Department of Public Health, University of Turku and Turku University Hospital, Turku, Finland; 6 Department of Epidemiology and Public Health, University College London Medical School, London, United Kingdom; 7 Department of Behavioral Sciences, University of Helsinki, Helsinki, Finland; Universidad Peruana Cayetano Heredia, Peru

## Abstract

**Background:**

While hypertension is a common and treatable health problem, adherence to antihypertensive medication remains a challenge. This study examines the hypothesis that workplace social capital may influence adherence to antihypertensive medication among hypertensive employees.

**Methodology/Principal Findings:**

We linked survey responses to nationwide pharmacy records for a cohort of 3515 hypertensive employees (mean age 53.9 years, 76% women) who required continuous antihypertensive drug therapy (the Finnish Public Sector study). A standard scale was used to measure workplace social capital from co-workers' assessments and self-reports in 2000–2004. Non-adherence to antihypertensive medication was determined based on the number of days-not-treated at the year following the survey using comprehensive prescription records. Negative binomial regression models were conducted adjusting for socio-demographic characteristics, duration of hypertension, behaviour-related risk factors, and co-morbid conditions. The overall rate of days-not-treated was 20.7 per person-year (78% had no days-not-treated). Higher age, obesity, and presence of somatic co-morbidities were all associated with better adherence, but this was not the case for co-worker-assessed or self-reported workplace social capital. The rate of days-not-treated was 19.7 per person-year in the bottom fourth of co-worker-assessed workplace social capital, compared to 20.4 in the top fourth. The corresponding rate ratio from the fully-adjusted model was 0.95 (95% confidence interval (CI) 0.58–1.56). In a subgroup of 907 new users of antihypertensive medication this rate ratio was 0.98 (95% CI 0.42–2.29).

**Conclusions/Significance:**

We found no consistent evidence to support the hypothesized effect of workplace social capital on adherence to drug therapy among employees with chronic hypertension.

## Introduction

Hypertension is an increasingly common health problem affecting currently one billion people worldwide [1]. Although effective medicines are available to control high blood pressure, adherence to treatment remains a major problem [2]. The extent of hypertensive patients who adhere to treatment (i.e. take medications as prescribed) [2], [3] is estimated to be 50% to 90% [4]–[9]. Lack of treatment adherence results in suboptimal blood pressure control, adverse cardiovascular outcomes, and health care costs that could have been avoided [2], [4], [10], [11]. Researchers have identified several correlates of adherence, including patient characteristics, the quality of the patient-clinician relationship, severity of disease, access to health care, and treatment regimen [2]–[4], [12]. Recent studies also suggest that social support may facilitate treatment adherence [2], [5], [13].

An important extension of the evidence linking social relationships to health outcomes is the growing literature on social capital and health. Social capital is defined as the features of social structures which act as resources for individuals, including interpersonal trust and norms of reciprocity and mutual aid [14]–[16]. Both low social capital and uncontrolled blood pressure have been linked to cardiovascular disease morbidity and mortality[11], [17], [18]. However it is unclear whether poor adherence to antihypertensive medication therapy could be one of the potential mechanisms linking low social capital to adverse cardiovascular outcomes.

In theory, social capital may influence medication adherence through: (a) the provision of effective social support networks for the exchange of health promoting information and access to resources outside the individual's own network [19]–[21]; (b) social engagement in a meaningful social context that promotes positive psychological states to enhance motivation for self-care and appropriate health service utilization [22], [23]; and (c) shared norms and values around health-related behaviours [24], [25]. To date, at least two studies have examined the relationship between social capital and the use of antihypertensive medication [26], [27]. However, both of these studies used self-reports to assess exposure and outcome and were thus subject to common method bias. Furthermore, the studies assessed social capital via social participation in the community among working and retired people. It can be argued that for working populations spending an increasing amount of their time at work, workplace may also represent a meaningful source of social capital [28], [29]. However, we are not aware of any previous studies examining the hypothesis that social capital in work context may promote adherence to antihypertensive drug therapy.

The aim of the present study was to examine the hypothesis that workplace social capital may influence adherence to antihypertensive medication in a prospective cohort study of 3515 hypertensive men and women who responded to a survey of workplace social capital. We linked their responses to nationwide pharmacy records of filled prescriptions for antihypertensive medication and followed up participants for one year to examine the rates of days-not-treated as an objective indicator of non-adherence to treatment.

## Methods

### Ethics statement

The Ethics Committee of the Finnish Institute of Occupational Health approved the study.

### Study design

We used data from the Finnish Public Sector study, which includes the entire personnel in the municipal services of 10 towns and within 6 hospital districts in Finland [30]. For this study, we included 66,418 employees who responded to a survey of workplace social capital in 2000–2002 and/or 2004 (a total of 79% of the eligible employees responded at least once). In case of repeat responses, the more recent one was used in the analysis. Personal identification numbers were used to link all study participants to national prescription and health registers from 1994 through 2005.

Of the respondents, we identified those who had physician-verified hypertension during the survey year, and who were alive at least one year after the survey year (total n = 4538). We excluded all 896 employees of one town for whom prescription data were not available because their medication costs were covered by the employer, 120 participants due to missing data on workplace social capital, and 7 participants who had died before the beginning of the 1-year follow-up which ran from January 1 following the survey year. Thus, the final analytic sample comprised 3515 hypertensive employees (76% women, mean age 53.9 years, range 22–66).

### Case ascertainment for hypertension

Hypertension and its duration were defined based on entitlement to special reimbursement. In Finland, the National Health Insurance administered by the Social Insurance Institution (SII) covers all permanent residents regardless of age, wealth, or employment status. Residents with chronic and severe hypertension are eligible for lower copayments for antihypertensive drugs. Currently, 72% of the costs of medicines are reimbursed after a fixed deductible by purchase with annual maximum copayments of around 600 Euros, provided the person is listed in the Drug Reimbursement Register maintained by the SII. In order to receive special reimbursement, the patient must submit a doctor's certificate to SII stating the illness, and the medication needed to treat it. For hypertension, the current criteria are stricter than the guidelines for treatment of hypertension [11], and include documentation of repeated blood pressure measurements of ≥105 mmHg diastolic, or ≥95 mmHg diastolic with signs of complications or other cardiovascular risk factors, or ≥200 mmHg systolic. Special reimbursement can be granted only after 6 months of surveillance with lifestyle counselling followed by 6 months of drug therapy.

### Workplace social capital

Workplace social capital was assessed with an 8-item self-assessment scale designed to measure social capital in the workplace [31]. Using a 5-point Likert-scale, the participants assessed workplace social capital, defined as the shared values, attitudes, and norms of trust and reciprocity as well as practices of collective action in their work unit [31]. As previously [32], [33], we assessed social capital in two alternative ways: (a) using each individual's own assessment, and to minimise subjectivity bias, (b) summing up the assessment of co-workers, but excluding the individual's own assessment. We calculated the mean of the individual items to construct a scale ranging from 1 to 5 with higher score indicating high social capital. Prior psychometric evaluation in the Finnish Public Sector Study has demonstrated the scale to have high internal consistency (Cronbach's alpha 0.88) and r_wg_ index (0.88) indicating significant within-unit agreement [31].

### Assessment of adherence to antihypertensive medication

Adherence to antihypertensive medication was assessed from filled prescriptions for antihypertensive drugs recorded in the Drug Prescription Register. The automated pharmacy dispensing data are operated under a single system and cover virtually all filled out-patient prescriptions from pharmacies in Finland. Pharmacy refill data of antihypertensive drugs were extracted for the year following the survey year, and included a listing of all antihypertensive prescriptions filled, the dispensing date, the quantity dispensed in Defined Daily Dosages (DDDs), and the drug class according to the WHO Anatomical Therapeutic Chemical (ATC) classification for codes C02 (antihypertensives), C03 (diuretics), C07 (beta blockers), C08 (calcium channel blockers), and C09 (ACE inhibitors). DDD is a globally unified measurement unit, defined as the assumed average maintenance dose per day for a drug used for its main indication in adults [34].

We used a refill-sequence model of adherence to quantify the duration of treatment [35]. Thus, we measured the total duration of consecutive refills, using 100 DDDs as a maximum for a refill (up to 3 months supply of medications can be reimbursed during a single transaction in the Finnish reimbursement legislation) and added 50 days to all refills to prospectively fill the gaps between subsequent purchases [35], [36]. This grace period for the gap in medication implies that the patients treated with half of the defined daily dose are correctly assigned as having 100 days of treatment. We then calculated (with this model) the length of the gaps, i.e. days-not-treated, between subsequent prescriptions in the sequence of all refill dates, assuming that an individual has not discontinued drug therapy at a given point in time if his/her dispensed supply from the last redemption has not elapsed or if it elapsed within the last 50 days. We excluded periods of inpatient hospital admissions during which the medication is provided by the hospital. The outcome, days-not-treated, indicated non-adherence to drug therapy and was treated as a continuous variable in the analyses [37], [38].

### Covariates

Participants' age, sex, socioeconomic status (SES), type of job contract (permanent vs. temporary), type of employer (municipality vs. hospital), and geographic area (Southern, Middle or Northern Finland, based on the location of the workplace) were derived from employers' registers. SES was divided into three categories according to the occupational classification of Statistics Finland, i.e. upper grade non-manual workers (e.g. head nurses, teachers), lower grade non-manual workers (e.g. practical nurses, technicians), and manual workers (e.g. cleaners, maintenance workers). Marital status (married or cohabiting vs. other) was derived from survey responses. Standard questionnaires were used to assess behaviour-related risk factors: obesity (body mass index≥30, calculated as weight in kilograms divided by height in meters squared), smoking (current vs. ex- or never), excess alcohol use (>210g/wk) [39], and physical inactivity (<2 MET-hours/wk, MET = metabolic equivalent tasks) [40].

The presence of co-morbid physical illnesses was based on documentation of the condition in the SII, the National Hospital Discharge Register, or the Finnish Centre for Pension for entitlement to special reimbursement for the costs of medication, sickness absence or disability pension or the main diagnoses of hospital discharge. These conditions included diabetes mellitus, heart failure, coronary heart disease, cardiac arrhythmia, and cerebrovascular diseases. Notifications of diagnosed cancer were obtained from the Finnish Cancer Registry which compiles all cancer notifications in Finland. Co-morbid conditions were encoded as ‘yes’ if any data on pre-existing disease was found during four years preceding the follow-up. Co-morbid depression was identified based on documentation of any of the following: 1) long term (>90 days) work disability (sickness absence or disability pension), 2) hospital admission due to depression, 3) pharmacy records of filled prescriptions of at least 30 defined daily dosages of antidepressants (ATC code N06) during any of the four years preceding the follow-up, or 4) if the participant responded as being diagnosed with depression in the survey.

### Statistical analysis

As of April 2003, pharmacy personnel have been allowed to switch to generic drug formulations to lower total prescription drug costs in Finland, additionally leading to smaller copayments to individuals, factor suggested to influence adherence [7]. We took this into account in the analyses by adjusting the models for the survey year. We examined the age- and survey-year-adjusted differences in rates of days-not-treated as a function of the cohort characteristics using analysis of variance. We calculated rate ratios (RR) and their 95% confidence intervals (CI) for the days-not-treated using negative binomial regression models to take into account the skewness of the outcome [41], allowing for overdispersion. We conducted the analyses separately for self-reported and co-worker-assessed social capital, adjusting for age, survey year, sex, socioeconomic position, marital status, type of job contract, type of employer, area of residence, duration of hypertension, behaviour-related risk factors, and co-morbid physical illness and depression at baseline. We tested whether sex or SES modified the association by adding the interaction terms sex*workplace social capital and SES*workplace social capital in the model of main effects, but we did not find any evidence of an effect modification (all p-values >0.05). Potential predictors of non-adherence were considered statistically significant at 95% confidence interval level (two-sided p<0.05).

### Sensitivity analyses

We conducted three sets of sensitivity analyses. First, as 80% of days treated is commonly used as a cut-point in adherence studies [3], [7], we tested the robustness of the findings by dichotomizing the outcome as less than 80% of days treated versus other. Using this binary outcome, we then conducted logistic regression models to calculate odds ratios (OR) for the risk of non-adherence associated with self-assessed and co-workers' assessment of workplace social capital. Second, because the effects of workplace social capital on health may vary by dimension [42], we examined the vertical (i.e. employees' relations with their employers and supervisors) and horizontal (i.e. social contacts, cooperation and trust in relation to co-workers) components of workplace social capital separately. We determined the vertical or horizontal component of workplace social capital as in our previous study [43], divided the co-worker assessed and self-reported ratings into quartiles, and examined whether they were associated with non-adherence with negative binomial regression models. Third, we restricted the sample to 907 (77% women) new users of antihypertensive medication because long-term users may differ from new users in terms of baseline risk; there is also evidence to suggest that adherence reduces over time [4], [6]. We traced those who were newly granted special reimbursement for hypertension after responding to a survey in 2000–02 or 2004, followed their adherence to antihypertensive medication during the first year of treatment starting from the date of granting special reimbursement, and repeated the main analysis using negative binomial regression models adjusting for age and year of treatment, and then for all covariates.

All statistical analyses were conducted with SAS 9.13 statistical software (SAS Institute, Inc., Cary, NC, USA).

## Results

The baseline characteristics of the 828 hypertensive men and 2687 women are shown in Table 1. Of them, 14% were smokers and 30% obese, 14% had been diagnosed with physical illnesses, and 20% with depression before the adherence follow-up begun. Participants had been treated for hypertension for a mean of 9.0 years (range 1–33) as defined from the duration of entitlement to special reimbursement. The means of self-assessed and co-workers' assessed workplace social capital were 3.56 (standard deviation (SD) 0.78, range 1–5) and 3.57 (SD 0.39, range 1.38–5), respectively.

**Table 1:** Baseline characteristics and their association with non-adherence to antihypertensive medication in 3515 hypertensive employees, the Finnish Public Sector Study, 2000–2005.

		Days-not-treated	
Characteristic	N (%)	Rate per 1 person-year*	RR (95% CI)*
Sex			
Women	2687 (76)	20.4	1.00
Men	828 (24)	21.7	1.06 (0.73–1.55)
Socioeconomic status			
Higher non-manual	799 (23)	20.0	1.00
Lower non-manual	1843 (52)	22.0	1.15 (0.77–1.72)
Manual workers	871 (25)	18.6	0.92 (0.58–1.46)
Marital status			
Married	2638 (76)	20.7	1.00
Not married	822 (24)	21.8	1.07 (0.73–1.56)
Type of job contract			
Permanent	3190 (93)	20.2	1.00
Temporary	246 (7)	25.2	1.23 (0.65–2.33)
Type of employer			
Municipality	2238 (64)	19.7	1.00
Hospital	1277 (36)	22.4	1.13 (0.80–1.59)
Geographical area			
Southern Finland	1919 (55)	20.7	1.00
Middle Finland	869 (25)	16.2	0.74 (0.50–1.10)
Northern Finland	727 (21)	26.0	1.20 (0.79–1.83)
Smoking			
No	2919 (86)	21.0	1.00
Yes	481 (14)	21.2	1.00 (0.63–1.60)
BMI ≥30			
No	2367 (70)	24.2	1.00
Yes	1034 (30)	12.4	0.51 (0.36–0.72)
Heavy alcohol use			
No	3097 (89)	21.4	1.00
Yes	398 (11)	15.1	0.68 (0.41–1.13)
Physical inactivity			
No	2251 (65)	22.7	1.00
Yes	1228 (35)	17.4	0.75 (0.53–1.04)
Co-morbid physical illness†			
No	3029 (86)	21.9	1.00
Yes	486 (14)	13.3	0.59 (0.37–0.94)
Co-morbid depression			
No	2804 (80)	19.8	1.00
Yes	711 (20)	24.3	1.24 (0.84–1.85)
Age (years, mean, SD)	53.9 (6.6)		0.86 (0.77–0.96)**
Duration of hypertension (years, mean, SD)	9.0 (6.3)		1.07 (0.94–1.22)**

RR; rate ratio, CI; confidence interval, SD; standard deviation.

*Adjusted for age and survey year.

**per 5-year increase.

† Diabetes mellitus, coronary insufficiency, coronary heart disease, cardiac arrhythmias, cerebrovascular disease.

The overall rate of days-not-treated was 20.7 per person-year (range 0–365 days). A total of 78% of the participants had no days-not-treated according to the Drug Prescription Register.

We did not find statistically significant differences in the rate of days-not-treated between men and women, by marital status, type of job contract or employer, residential area, or between socioeconomic groups (Table 1). Smoking, physical inactivity and heavy alcohol use were not associated with adherence either. In contrast, each 5-year increase in age reduced the rate of days-not-treated by 14% (RR 0.86, 95% CI 0.77–0.96). Obesity and the presence of somatic co-morbidity were also associated with an improved adherence: adjusted for age and survey year, rate ratios for days-not-treated were 0.51 (95% CI 0.36–0.72) for the obese and 0.59 (95% CI 0.37–0.94) for the participants with somatic co-morbidities compared to employees without these conditions.

Table 2 shows that neither co-worker assessed nor self-reported workplace social capital was associated with adherence to antihypertensive medication at 95% confidence level. The rate of days-not-treated was 19.7 per person-year in the bottom fourth of co-worker-assessed workplace social capital, compared to 20.4 in the top fourth. The corresponding rate ratio, adjusted for socio-demographic characteristics, duration of hypertension, behaviour-related risk factors, and co-morbid conditions was 0.95 (95% CI 0.58–1.56). For self-reported workplace social capital, the rate of days-not-treated was 23.2 among employees in the lowest quartile, whereas it was 21.7 for those reporting the highest levels. The corresponding rate ratio from the fully-adjusted model was 1.17 (95% CI 0.72–1.92).

**Table 2:** Association between workplace social capital and non-adherence to antihypertensive medication in 3515 hypertensive employees, the Finnish Public Sector Study, 2000–2005.

		Days-not-treated		
Workplace social capital	Rate per 1 person-year	Model 1RR (95% CI)*	Model 2RR (95% CI)*	Model 3RR (95% CI)*
**Self-assessed**				
1 (low)	23.2	1.03 (0.65–1.65)	1.13 (0.70–1.83)	1.17 (0.72–1.92)
2	19.2	0.82 (0.52–1.30)	0.91 (0.57–1.45)	0.85 (0.53–1.38)
3	18.9	0.87 (0.54–1.39)	0.94 (0.58–1.52)	1.04 (0.62–1.73)
4 (high)	21.7	1.00	1.00	1.00
**Co-workers**' **assessment**				
1 (low)	19.7	1.00 (0.63–1.57)	0.97 (0.60–1.57)	0.95 (0.58–1.56)
2	21.2	1.05 (0.66–1.66)	1.04 (0.64–1.67)	0.92 (0.56–1.52)
3	21.4	1.04 (0.66–1.65)	0.96 (0.60–1.55)	0.80 (0.48–1.32)
4 (high)	20.4	1.00	1.00	1.00

*RR; rate ratio, CI; confidence interval.

Model 1 adjusted for age and survey year.

Model 2, as Model 1, additionally adjusted for sex, socioeconomic and marital status, type of job contract, type of employer, geographical area, and the duration of hypertension.

Model 3, as Model 2, additionally adjusted for smoking, excess alcohol use, obesity, physical inactivity, and co-morbid physical illness and depression.

The results from the sensitivity analysis were consistent with the main findings. Modelling the risk of less than 80% of days-treated as the outcome and adjusting for all the covariates showed that employees in the lowest quartile of co-worker assessed workplace had no excess risk of non-adherence (OR 1.06, 95% CI 0.66–1.38) compared to those in the highest quartile. For the lowest levels of self-reported workplace social capital, the odds ratio from the fully-adjusted model was 1.16 (95% CI 0.81–1.66). Similarly, neither co-worker assessed nor self-reported vertical or horizontal component of workplace social capital were associated with adherence; the rate ratios varied between 1.00 and 1.12, and all 95% confidence intervals included unity.

In the subcohort of 907 new users of antihypertensive medication, the overall rate of days-not-treated was 18.7 per person-year during the first year of treatment when entitled to special reimbursement for the costs of medication. Table 3 shows that among these new users of antihypertensive medication there was no excess risk of non-adherence associated with co-worker assessed or self-reported workplace social capital. The rate ratio of days-not-treated was 0.98 (95% CI 0.42–2.29) for low vs. high co-worker assessed workplace social capital and 1.43 (95% CI 0.60–3.40) for low vs. high self-reported social capital.

**Table 3:** Association between workplace social capital and non-adherence to antihypertensive medication in a subcohort of 907 employees with incident hypertension, the Finnish Public Sector Study, 2000–2005.

	Days-not-treated		
Workplace social capital	Rate per 1 person-year	Model 1RR (95% CI)*	Model 2RR (95% CI)*
**Self-assessed**			
1 (low)	24.5	1.56 (0.71–3.44)	1.43 (0.60–3.40)
2	20.7	1.38 (0.64–3.00)	1.27 (0.54–2.94)
3	13.4	0.80 (0.78–1.69)	0.92 (0.40–2.13)
4 (high)	18.1	1.00	1.00
**Co-workers**' **assessment**			
1 (low)	18.4	1.04 (0.48–2.24)	0.98 (0.42–2.29)
2	21.0	1.23 (0.57–2.64)	1.08 (0.47–2.47)
3	15.3	0.86 (0.40–1.85)	0.70 (0.31–1.60)
4 (high)	18.1	1.00	1.00

*RR; rate ratio, CI; confidence interval.

Model 1 adjusted for age and treatment year.

Model 2, as Model 1, additionally adjusted for sex, socioeconomic and marital status, type of job contract, type of employer, geographical area, duration of hypertension, smoking, excess alcohol use, obesity, physical inactivity, and co-morbid physical illness and depression.

## Discussion

In this cohort of 3500 hypertensive employees, we found no consistent evidence to support the hypothesis that workplace social capital would be associated with adherence to antihypertensive medication. This was true for all and new users of antihypertensive medication, for self-assessed and co-workers' assessment of workplace social capital, and for its vertical and horizontal components.

Our results are in line with Johnell et al. who found no robust association between social participation in the community and adherence to antihypertensive medication among the elderly [26]. Similarly, Merlo et al. found no neighbourhood effect of social participation on self-reported antihypertensive medication use among women [27]. In our study, low self-reported social capital was non-significantly associated with non-adherence, whereas the association of co-worker-assessed social capital and adherence was practically null. Given that we had sufficient power to detect a meaningful association between social capital and adherence, these null results suggest that workplace social capital does not explain non-adherence to pharmacotherapy in hypertensive working populations.

It is important to consider alternative explanations for our results. The American Society of Hypertension and empirical studies have highlighted that factors related to the health care system are undervalued as contributors to (in)sufficient adherence, as access to health care services may vary among health care systems leading to cost-related non-adherence [4], [44]. In Finland, all citizens have unrestricted access to health services, including partial or complete reimbursement of purchased medicines. In these circumstances it may be that social capital in the workplace promotes regular check-ups and help seeking in the first place rather than continued adherence to medication [22], [43]. Once a patient has commenced long-term therapy, it is possible that other characteristics, such as age, overall life style and, psychological traits, may affect treatment adherence, as demonstrated in a previous study in this cohort [41].

Imprecise measurement of the exposure or the outcome may contribute to null findings. It is unlikely that the social capital measure is subject to appreciable measurement error because we also assessed co-workers' perceptions of workplace social capital in the same work unit, thus reducing the possibility of common method and subjectivity biases related to self-report. Furthermore, the workplace social capital measure has successfully predicted other health outcomes, such as depression, in this dataset [32], [33].

By and large, the measurement of adherence in hypertension is problematic because no direct measures, such as biological markers measured from the blood, are available [3]. We did not use self-reports of adherence which are subject to recall bias and social desirability with the tendency to overestimate adherence [6], [45]. Comprehension of monitoring of adherence as in randomised controlled trials may itself enhance adherence [9]. Measuring adherence objectively from pharmacy refill data are considered highly accurate and more complete than medical records and information elicited from questionnaires [46]. In our study, the rates of adherence observed were in line with previous studies from the UK [5], US [7], [8], and Spain [9] reporting days covered rather than using an arbitrary cut-point to determine adherence. Furthermore, we found, in agreement with previous studies, that older age, obesity, and co-occurrence of cardiovascular co-morbidities were each associated with better adherence [47], [48]. Patients with multiple cardiovascular risk factors may be more motivated to adhere with their therapy or have more severe of hypertension [47], [48].

Limited variation in the exposure may lead to an underestimation of associations and potentially contribute to false null findings. All employees in our study were working in the service of public hospitals or municipalities. It is possible that the workplace culture in these public sector workplaces is more similar than in other types of workplace (for example in private for-profit companies), resulting in reduced variation in workplace social capital. However, the range and standard deviation of the social capital measures suggest that limited variation may not be a major problem in these data

Low statistical power can prevent detection of small effects. Our study was based on a relatively large sample and the absolute differences in non-adherence days between social capital groups were all within 6 days per year. This suggests that there was no clinically meaningful effect of workplace social capital on adherence behaviour. Still, based on the results of this study, we cannot refute the possibility that social capital in other contexts might be associated with adherence to antihypertensive medication or treatment adherence in other diseases.

### Strengths and limitations

We employed data from a large cohort of workers with physician-verified hypertension and need for continuous antihypertensive medication, successfully linked to a comprehensive population level prescription register to enable a 12-month prospective follow-up. All participants were covered by the Finnish National Health Insurance, thus avoiding selection bias due to insurance coverage or varying time of insurance coverage which is possible in commercial health insurance plans. We investigated both prevalent and new users because barriers to good adherence may differ for newly-diagnosed patients versus those with long-standing treatment for hypertension [6], [7]. We used a psychometrically validated multi-item measure to assess workplace social capital. We measured medication adherence objectively from pharmacy refill data. Rates of refilled prescriptions are considered valid as measures of gaps in medication supply within a closed pharmacy system provided that the refills are measured at several points in time [3], [49].

However, the results need to be interpreted in the light of several limitations. First, pharmacy dispensing records provide an indirect measure of adherence and we could not ascertain whether the medication was actually taken. Second, the participants were covered by the Finnish National Health Insurance which may compromise generalisability of the findings to uninsured workers. Third, qualifying for special reimbursement required blood pressure levels of Stage II hypertension or Stage I with other cardiovascular risk factors or co-morbidities [11]. Hence, we do not know whether workplace social capital contributes to adherence in milder cases of hypertension. Fourth, we assessed workplace social capital at baseline only, and we did not assess changes in social capital in relation to changes in adherence behaviour. Fifth, when prescription registries are used to define episodes of medication use, apparent gaps in treatment can occur. By filling the gaps, we avoided bias from embedded gaps due to a lower dose than DDD or tablet splitting; however, we could still have misclassified participants with terminal gaps, i.e. discontinuing the medication without finishing the supply [50]. If the 50 days we used for filling the gaps is too long a period, we have counted those non-adherent as adherent; conversely, if the period is too short, we have missed individuals who continued taking the medication, i.e. who were adherent. Non-differential misclassification of the outcome, which can be assumed to be the case with the Prescription Register, is likely to bias the measures of associations towards the null. Finally, there may be a tendency of those employees whose adherence is poor to develop complications of high blood pressure and to leave employment [10], [50]. However, our follow-up covered both employed and unemployed population, reducing the healthy worker effect related to occupational cohorts [51].

### Conclusions

In this paper, we report null results of the relationship between workplace social capital and adherence to antihypertensive medication. Reporting null results may provide balance for the social capital research area not to produce publication bias and not to show social capital in a too favourable light. We wish not to guard research away from studying social capital in workplaces, but instead, encourage comparative work. To our knowledge, this is the first and only investigation on this topic in the work context to date. As the meaning of workplace social capital may differ by cultural background or branch of industries, further studies in other cohorts and contexts are needed to refute the overall hypothesis that social capital is associated with adherence to antihypertensive medication.
